# Notes on “Euphorbia Prostrata” as an Antidote to the Poison of the Rattlesnake

**Published:** 1861-03

**Authors:** B. J. D. Irwin

**Affiliations:** Assist. Surgeon U. S. Army


*Notes on "Euphorbia Prostrata" as an Antidote to the Poison of the Rattlesnake.* By B. J. D. Irwin, M.D., Assist. Surgeon U. S. Army.

During the summer of the last year my attention was attracted through the public press to the unusually large number of persons reported as having lost their lives from the bite of the rattlesnake. Many fatal cases were recorded from almost all parts of the United States, but the greater number appeared to have occurred in the South and Southwestern regions. Knowing how abundant the several varieties of *Crotalus* are in this region of country, and never having heard of any case of death from the poison of this reptile amongst the inhabitants, I was led to make inquiries of the Mexican population as to what protective measures they resorted to in case of injury from the rattlesnake or the many other poisonous creatures that infest the country. After many inquiries among the natives, Mexicans and civilized Indians, I learned that, although injuries from poisonous reptiles were very common among them, they had an efficacious antidote against the poison of the rattlesnake and other noxious reptiles. The remedy resorted to I found to be what they designate as " *Gollindrinera* (*Anglice*, swallowwort), an herb of very frail, delicate appearance, found growing abundantly throughout the southern portion of Texas, New Mexico, Arizona, and Sonora. A specimen forwarded through the Smithsonian Institution to Professor Torry was pronounced by him to be "*Euphorbia Prostrata*," a plant belonging to a family entirely different from that of the swallowwort. It grows plentifully in dry, hard, sandy places, especially in roadways, farm-yards, pathways, and in a hard, compact, gravelly soil, and has a frail, delicate appearance, resembling in its external character the gold-thread (*Coptis trifolia*), with long, filiform, reddish stems, that spread and interlace with each other. Leaves, petaloid, obcordate, regular, opposite, of a deep-green color, and varying in length from three to five lines. Flowers, axillary and very small, white, with dark-purple throat; sepals four, petals four,pentandria monogynia. Root quite large, dark-brown color, and possessing an abundance of milky juice, which pervades all parts of the plant; taste insipid; odourless. Flowers from April till November.

Having heard much of the rare virtues of this plant, I determined to satisfy myself of its properties by proper investigation, and, after a series of experiments, carefully performed, I had the gratification to find that its qualities as an antidote to the poison of the rattlesnake are equal to that now used and known as "Bibron's Antidote." The experiments were made on many dogs, and extended through a period of several months, with like satisfactory results. The medicinal properties of the plant I found to reside in the milk-like juice of the stem, root, and leaves; but that usually used was the fresh juice extracted from the plant by bruising it in an iron mortar, and diluting it with a considerable quantity of water. As to its *modus operandi*, I am not prepared to offer an explanation; its physiological effects are emetic and cathartic, when given in large quantity. As it would be tedious to give the history of all the experiments made, I will confine myself to giving the notes of one or two cases in detail.

*Experiment* 1. August 16, 1859. At 11 o'clock A. M. a full-grown slut was subjected to the bite of a large rattlesnake (Crotalus confluents), which was very much infuriated and inflicted a severe wound at the root of one of the ears, where it hung by its fangs for several moments. The dog immediately became greatly agitated, very restless, and whined most piteously. Ten minutes after, she was unable to stand up; respiration hurried; drunken appearance, with bloodshot eyes; involuntary evacuation of urine and feces; no swelling about the injured part. Administered four fluid ounces of the watery juice of the freshly-gathered "Gollindrinera," and applied some of the bruised plant to the wound. Two minutes afterwards the dog got up and walked about, with only a slight staggering gait.

12 o'clock M. Respiration is still very hurried and difficult; restless; eyes very red, but drunken appearance is rapidly diminishing; no swelling of the injured part; dog is walking about, but with an unsteady, rolling gait; disposition to vomit. Comes when called, and evinces pleasure on be caressed. Repeated the dose.

1 o'clock P. M. Is almost well; walks with firmness, and appears to have ceased suffering. Between two and three o'clock vomited freely, first food, and afterwards some dirty-looking watery fluid, consisting in part of the remedy administered. About eight ounces were given, of which two ounces were pure juice. The dog partook of food and drink about five o'clock, and was running about, in seemingly good health, at evening time.

*Expt.* 2. August 20, 1860. A large dog was bitten on the nose by a rattlesnake having nine rattles. Thirty minutes afterwards he was unable to stand up, and apparently insusceptible of external impression; respiration hurried; eyes congested; general appearance of speedy death; head and neck enormously swollen. Four fluidounces of the watery juice of the "Gollindrinera" were poured down his throat, and a cold epithem of the bruised plant applied over the swollen parts. Within fifteen minutes he got up and walked about with a listless air and unsteady gait. Two hours afterwards the condition was vastly improved; swelling greatly diminished, intelligence restored, respiration almost natural, and the eyes and expression had assumed almost their natural characters. Repeated the medicine in the same quantity.

By evening the dog was entirely well, save some swelling, which disappeared completely by next morning.

The phenomena manifested in all the other cases, excepting one, were similar in every respect to the foregoing. The exceptional case proved fatal to a *very old little* dog, to which I did not give the antidote until he was apparently dead. He resuscitated shortly after taking the remedy, but death by asthenia took place before the remedy could produce its effects. In one particular dog no constitutional symptoms could be produced by the poison of the rattlesnake; although she was severely bitten by two snakes at separate intervals, and I inserted some of the poison from a fang into a wound made for its reception, nevertheless nothing more than local swelling and pain could be induced in her system. I cannot account for the immunity enjoyed by that animal, as the same reptiles caused deadly poisoning in other dogs.

From the universal dissemination of this plant throughout the south-western portion of the United States and Mexico, I believe a knowledge of its therapeutic properties will prove of much importance to the public, as it grows plentifully through the whole year; its preparation is simple and its administration is unattended with danger to the animal economy. I have learned from the Mexicans of many cases of poisoning by the rattlesnake-bite cured by its use, and in no case has it failed to cure, or has it done injury to the patient.

In my reading, I find that other varieties of Euphorbiaceæ enjoy a reputation as remedies against the poisons of noxious reptiles. The *Euphorbia capitata*, *E. corollata*, *E. palustris* and *E. villosa*, are somewhat celebrated in southern countries as specifics against the poison of venomous serpents and the bite of rabid animals. One of the strongest arguments that I may adduce in favour of the *Euphorbia prostrata* is, that amongst the Mexican population of Arizona and Sonora, who are frequently subjected to piosonous wounds from the rattlesnake, coral snake, "vinegrilla"

(*Thelyphonus*), scorpion, centipede, tranantula, and a host of other hideous creatures, in no instance does the injury result fatally, as they resort at once to their specific, ycleped "*Gollindrinera*," which never fails to produce a sure and speedy cure. In the hands of unscientific persons, I look upon it as an invaluable specific, possessing many advantages over that of M. Bibron, as it can be readily procured, easily prepared, and administered by any person, free from the many difficulties and disadvantages attending the preparation, preservation and administration of M. Bibron's valuable remedy. —*American Journal of Medical Sciences.*

Fort Buchanan, Arizona, Sept. 7, 1860.

